# Biofilm Formation and Spore‐Mediated Persistence of *Clostridium perfringens* in Meat and Poultry Processing Environments and Their Implications for Control Strategies

**DOI:** 10.1111/1750-3841.71193

**Published:** 2026-06-18

**Authors:** Md Anamul Hasan Chowdhury, Chowdhury Sanat Anjum Reem, Md. Ashrafudoulla, Hae Jung Yoon, Sang‐Do Ha

**Affiliations:** ^1^ Department of Food Safety and Regulatory Science Chung‐Ang University Anseong‐si Gyeonggi‐do Republic of Korea; ^2^ GreenTech‐Based Food Safety Research Group, BK21 Four Chung‐Ang University Anseong Gyeonggi‐do Republic of Korea; ^3^ Department of Food Science and Technology The Ohio State University Columbus Ohio USA

**Keywords:** antimicrobial strategies, biofilms, *Clostridium perfringens*, food safety, meat and poultry processing, spores

## Abstract

*Clostridium perfringens* (*C. perfringens*) biofilms pose a persistent challenge in meat and poultry processing environments due to their structural resilience, spore‐mediated survival and toxin‐associated virulence. These biofilms readily develop on food‐contact surfaces under typical processing conditions including organic residue accumulation, temperature fluctuations, and localized anaerobic niches, leading to increased tolerance to sanitation and thermal treatments. Mechanistically, biofilm resilience in *C. perfringens* is governed by the integration of sporulation processes, quorum sensing–regulated gene expression and extracellular polymeric substance (EPS) matrix formation, which collectively enhance stress tolerance, limit antimicrobial penetration, and facilitate persistence under fluctuating environmental conditions. The interaction between spore formation and EPS architecture further promotes survival during thermal processing and enables rapid re‐establishment of biofilms following sanitation. This review synthesizes current knowledge on the formation and persistence of *C. perfringens* biofilms, key environmental drivers in meat and poultry processing systems and the mechanistic basis of their stress resistance and survival strategies. It also critically examines how these mechanisms influence the efficacy of existing intervention strategies. It further evaluates the limitations of conventional control strategies and highlights emerging approaches for biofilm prevention and control, including food‐grade antimicrobials, surface engineering, enzymatic disruption, and microbiome‐based interventions, with emphasis on their modes of action and applicability in industrial settings. Overall, this review provides a mechanistic and systems‐level perspective to support the development of more effective biofilm control strategies in meat processing environments.

## 1 Introduction

*Clostridium perfringens* is a spore‐forming, obligate anaerobic, Gram‐positive foodborne pathogen associated with meat and poultry products and is responsible for millions of cases of foodborne illness globally each year (Grenda et al. 2023). The organism is particularly problematic in meat processing systems because it can survive multiple environmental stresses encountered during production, including heat treatment, chilling, and oxygen fluctuations (Cooper et al. 2013). This resilience is largely attributed to its ability to form highly resistant endospores and rapidly germinate under favorable conditions (Xiao et al. 2025). In addition, *C. perfringens* can form persistent biofilms on food‐contact surfaces, allowing the pathogen to survive sanitation procedures and recontaminate products during processing (Oliulla, Mizan, Ashrafudoulla, et al. 2024). These biofilms function as protective reservoirs that facilitate bacterial survival, postprocessing contamination and rapid regrowth when temperature abuse occurs, making effective control of this pathogen a continuing challenge for the meat and poultry industry (Mazaheri et al. 2021). In food safety systems, *C. perfringens* represents a biological hazard due to its toxigenic and spore‐forming characteristics, whereas the associated risk depends on the likelihood of contamination, survival during processing, temperature abuse, and subsequent consumer exposure (Koutsoumanis et al. 2023).

In *C. perfringens*, biofilm formation further enhances persistence by integrating vegetative cells with spores within an extracellular polymeric substances (EPS) matrix, which provides structural stability and protection from environmental stresses (Bhattrai et al. 2026). This matrix can enhance tolerance to heat, sanitizers, desiccation and mechanical removal, enabling pathogens to persist on processing equipment such as grinders, slicers, conveyor belts, chilling tanks and cutting boards. As a result, biofilms on food‐contact surfaces can act as continuous sources of contamination throughout multiple production cycles.

The risk of *C. perfringens* biofilm formation is further amplified by conditions commonly present in meat and poultry processing facilities. Organic residues from meat products provide nutrient‐rich microenvironments that promote bacterial attachment and growth (Zhu et al. 2025). These conditions are particularly favorable for *C. perfringens* due to its anaerobic metabolism and ability to proliferate in oxygen‐limited niches (McClane et al. 2012). Many processing steps, including evisceration, cutting, deboning, and postcook handling, expose equipment surfaces to moderate temperatures that support bacterial survival (L. Huang and Li 2020). In addition, repeated mechanical use of equipment often produces microscopic scratches and crevices that facilitate bacterial adhesion and biofilm development. Frequent interactions among workers, carcasses, tools and processing surfaces also increase the potential for cross‐contamination within processing lines (Coughlan et al. 2016). Together, these factors contribute to the persistence of biofilms that can resist conventional sanitation practices. Traditional biofilm control approaches, including thermal treatments, chlorine‐based sanitizers, quaternary ammonium compounds, and mechanical cleaning, often show limited effectiveness against mature biofilms. This limitation is particularly critical for *C. perfringens* due to the combined resistance of spores and biofilm‐associated cells. Furthermore, increasing regulatory restrictions, environmental concerns, and consumer demand for safer food production systems are driving the search for alternative control strategies. A deeper understanding of the biological mechanisms governing *C. perfringens* biofilm formation and persistence is therefore essential for developing effective intervention approaches.

While several previous reviews have addressed general biofilm formation in food systems or focused on other major pathogens, studies specifically integrating the unique spore‐forming capacity, anaerobic adaptability, and surface‐specific persistence of *C. perfringens* within meat and poultry processing environments remain limited. In particular, existing literature often treats biofilm formation, environmental drivers, and control strategies as separate topics, with limited emphasis on their interdependence within real processing lines. This fragmented approach limits the translation of mechanistic insights into effective and industry‐relevant intervention strategies.

Moreover, the unique physiological characteristics of *C. perfringens*, particularly its obligate anaerobic metabolism and spore‐forming capacity, are not sufficiently incorporated into current biofilm models. The interaction between sporulation processes and EPS matrix development, and how this interaction contributes to persistence, stress resistance, and sanitation failure in meat processing environments, remains underexplored. In addition, limited attention has been given to how processing‐specific factors, such as organic load, surface microstructure, and fluctuating redox conditions, influence these mechanisms and ultimately affect the performance of control strategies. To address this gap, this review adopts a conceptual framework in which *C. perfringens* biofilm formation is driven by the interaction of EPS, sporulation, and regulatory systems. EPS provides structural protection, spores ensure persistence under stress, and regulatory networks coordinate these processes in response to environmental conditions. This integrated perspective links biofilm development with survival and improves the mechanistic basis for targeted control strategies in meat processing environments.

This review aims to provide a comprehensive overview of the mechanisms underlying *C. perfringens* biofilm formation in meat and poultry processing environments and to evaluate current and emerging strategies for biofilm prevention and control. In contrast to previous reviews, this work specifically focuses on the organism‐level behavior of *C. perfringens* by linking its sporulation, anaerobic adaptation, and biofilm‐forming capacity to persistence in processing environments. Specifically, it integrates spore‐driven persistence mechanisms, processing‐environment‐specific drivers, and targeted control strategies into a unified framework relevant to industrial meat systems. Particular attention is given to factors influencing biofilm development on food‐contact surfaces and to potential interventions that can enhance microbial safety in modern meat processing systems.

## 2 Biology of *C. perfringens* Biofilms

**FIGURE 1:**
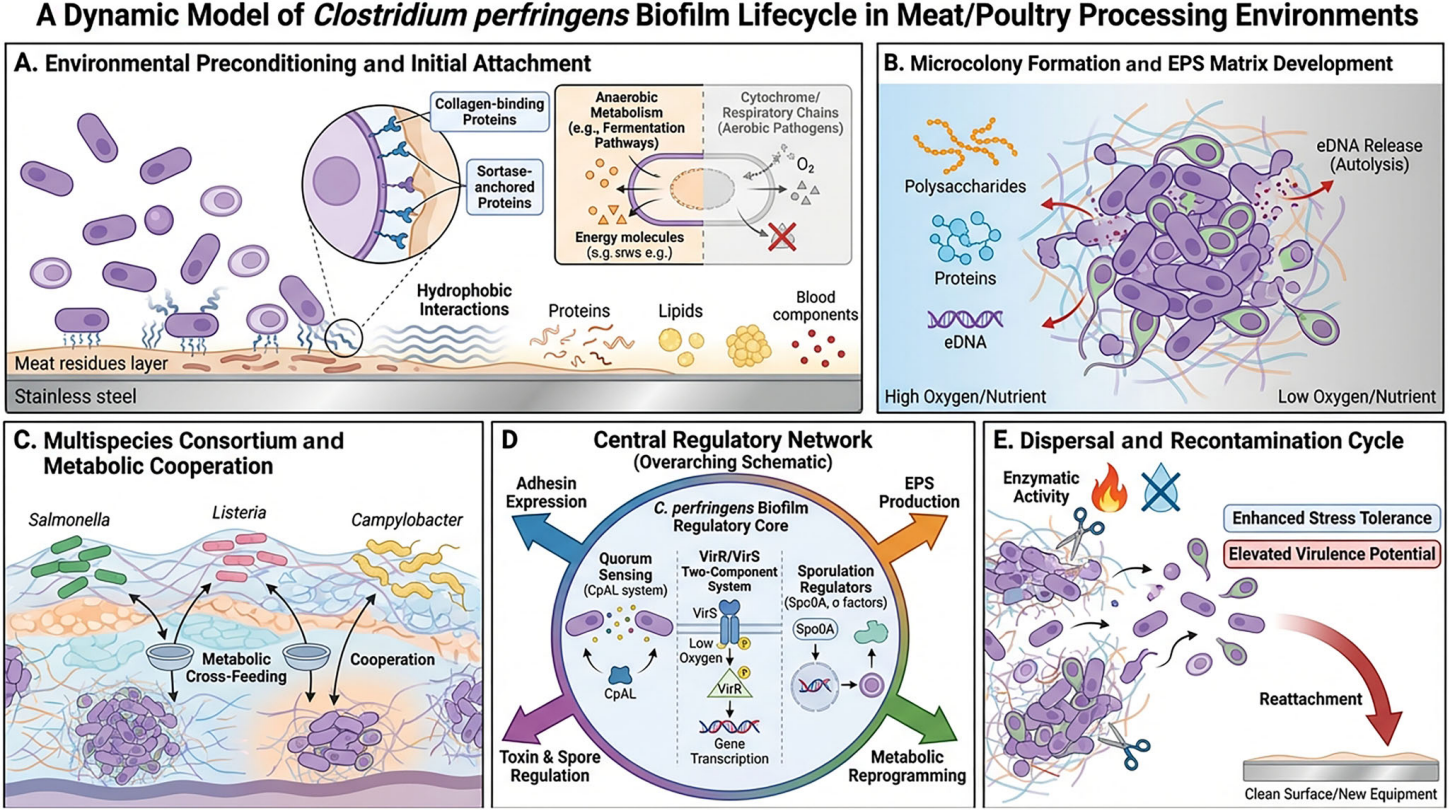
Integrated lifecycle, regulatory networks, and pathogen synergy of Clostridium perfringens biofilms in meat and poultry processing systems. The schematic illustrates the main stages of *C. perfringens* biofilm development on food‐contact surfaces. (A) Initial attachment to surfaces conditioned with organic residues such as proteins and fats. (B) Microcolony formation and maturation characterized by the production of extracellular polymeric substances (EPS). (C) Development of multispecies biofilms involving interactions with other foodborne pathogens, including *Salmonella* and *Listeria*. (D) Regulatory pathways influencing biofilm development, including quorum sensing, two‐component regulatory systems, and sporulation‐associated regulators. (E) Biofilm dispersal leading to the release of vegetative cells and spores, which contribute to contamination of new surfaces within processing environments.

Biofilm formation by *C. perfringens* represents a specialized survival strategy that is closely linked to its anaerobic metabolism and spore‐forming capacity, distinguishing it from many non–spore‐forming foodborne pathogens (Hu et al. 2021). These biofilms provide significant protection against environmental stresses such as thermal processing, cleaning regimens and fluctuating oxygen conditions encountered in meat and poultry environments (Araújo et al. 2024). Unlike typical Gram‐positive biofilm formers, *C. perfringens* integrates sporulation and vegetative growth within biofilm structures, enabling dual survival modes under stress conditions (Kaur and Dey 2023). These traits allow the organism to transition between vegetative growth, sporulation and biofilm‐associated states, enabling persistence across multiple processing cycles (Savard et al. 2025). This integrated lifecycle, combining biofilm formation with spore‐mediated resilience, represents a key challenge for control in meat processing environments (Samrot et al. 2021). Figure 1 summarizes the major stages involved in the biofilm lifecycle of *C. perfringens*.

### 2.1 Stages of Biofilm Formation

Biofilm development in *C. perfringens* follows a multistage process that is strongly influenced by anaerobic microenvironments, nutrient‐rich residues and dynamic redox gradients characteristic of meat processing systems (Bai et al. 2021). The organism's anaerobic metabolism and spore‐forming capacity further enhance persistence on food‐contact surfaces (Hu et al. 2021). Importantly, among environmental factors, nutrient availability and anaerobic conditions are the primary drivers of biofilm formation, while surface properties, redox fluctuations, and sanitation stress act as secondary but reinforcing factors influencing biofilm stability and maturation (Mahapatra and Ankri 2025). Initial cell attachment occurs on surfaces conditioned by meat residues such as fats, proteins, and blood components, which promote bacterial adhesion through hydrophobic interactions and surface‐associated adhesins. In *C. perfringens*, adhesion is further facilitated by spore surface structures and protein‐binding interactions that enhance attachment to organic‐rich substrates. Spores also contribute to early colonization due to their affinity for lipid‐ and protein‐rich substrates (Alzubeidi et al. 2018; Mehdizadeh Gohari, Navarro, et al. 2021). During early biofilm development, attached cells form microcolonies within anaerobic microenvironments created by organic residues and surface irregularities. These localized oxygen‐limited niches are particularly favorable for *C. perfringens*, supporting simultaneous vegetative growth and sporulation (Guzmán‐Soto et al. 2021).

Following attachment, bacteria produce EPS that form a structured matrix. The EPS of *C. perfringens* biofilms is a complex and heterogeneous network primarily composed of polysaccharides, proteins, extracellular DNA (eDNA), and lipids, each contributing to structural integrity and functional resilience (Rather et al. 2021). At the molecular level, eDNA contributes to structural stability and cell–cell adhesion, proteins function as enzymes and structural components, and polysaccharides form the primary scaffold of the matrix (Karygianni et al. 2020). In *C. perfringens*, the EPS matrix not only provides structural stability but also facilitates spore entrapment and protection against sanitizers and heat stress. (Di Martino 2018). Mechanistically, EPS acts as a diffusion barrier that limits sanitizer penetration, binds and neutralizes antimicrobial agents, and creates localized microenvironments with reduced oxygen and altered pH, which further enhance bacterial survival and stress tolerance (Panthi et al. 2024). Additionally, reduced metabolic activity of deeply embedded cells and the presence of dormant spores within the biofilm further decrease sanitizer susceptibility, allowing a subpopulation of cells to survive conventional disinfection procedures (Coppola et al. 2025).

As the biofilm matures, its matrix becomes heterogeneous, forming microgradients that support metabolic interactions with coexisting organisms, such as *Salmonella*, *Listeria*, and *Campylobacter* (Montanari et al. 2025). These multispecies interactions may further enhance biofilm resilience and complicate sanitation efforts (Olaimat et al. 2024). Specifically, multispecies biofilms can promote metabolic cooperation, where metabolic by‐products from one organism support the growth of another, and can provide physical protection that enhances persistence of *C. perfringens* in competitive environments. Additionally, increased structural complexity and EPS production in multispecies systems can lead to higher tolerance to disinfectants and environmental stress (Ren et al. 2024).

This process may involve enzymatic degradation of EPS and changes in gene expression triggered by environmental stress.

### 2.2 Key Regulatory Factors in Biofilm Formation

Biofilm formation in *C. perfringens* is regulated by genetic systems that respond to environmental signals. Key regulatory mechanisms include quorum sensing systems, two‐component regulatory systems and sporulation‐linked transcriptional regulators that coordinate biofilm development with environmental adaptation. (Vidal et al. 2015). The Agr‐like quorum sensing system of *C. perfringens* regulates toxin production and biofilm‐associated behaviors, including EPS synthesis and transitions between vegetative growth and sporulation (Obana et al. 2020). This system operates through signaling peptides that accumulate with increasing cell density and activate gene expression related to EPS production and biofilm maturation. This system plays a central role in coordinating population‐level responses, linking cell density with virulence expression and biofilm maturation (Zhang, Zhang, et al. 2025). The VirR/VirS two‐component regulatory system controls the expression of virulence and surface‐associated genes involved in colonization and environmental stress adaptation (Mehdizadeh Gohari, Li, et al. 2021). This regulatory pathway enables rapid response to environmental changes such as nutrient availability and stress exposure. Sporulation‐related regulators such as Spo0A and associated sigma factors contribute to biofilm persistence by linking sporulation with biofilm development (Put et al. 2024). Spo0A, in particular, functions as a master regulator that integrates environmental signals to determine the transition between vegetative growth and spore formation within biofilms (Zhang, Palma, et al. 2025). Importantly, these regulatory systems are functionally interconnected: Agr‐mediated quorum sensing primarily senses population density, which can influence VirR/VirS activation, while both systems converge on Spo0A‐dependent pathways to coordinate the switch between virulence, biofilm maturation, and sporulation states (Williams 2025). Thus, Agr acts as an upstream population‐density sensor, VirR/VirS functions as an adaptive environmental response regulator, and Spo0A integrates both signals to control long‐term developmental outcomes such as sporulation and biofilm persistence (Li and McClane 2020). This regulatory integration enables *C. perfringens* to rapidly respond to environmental fluctuations by shifting between growth, dormancy and biofilm‐associated states.

### 2.3 Role of Spores in Biofilm Persistence

Spores represent the most critical factor driving the persistence of *C. perfringens* biofilms in meat and poultry processing environments. Due to their highly resistant outer structures, spores strongly attach to hydrophobic and protein‐rich surfaces (Taylor et al. 2025). Their resistance to heat, desiccation, and chemical sanitizers allows them to survive standard cleaning procedures (Ban‐Cucerzan et al. 2025). Spores act as primary colonization units that survive sanitation and later germinate into vegetative cells (Xie et al. 2025). Within mature biofilms, EPS can embed spores, enhancing resistance to heat, desiccation and chemical sanitizers (Breitenbach et al. 2022). This physical entrapment within the EPS matrix further limits exposure to external stressors and contributes to prolonged survival. Entrapped spores can survive cleaning processes and later germinate when favorable conditions return, resulting in rapid recolonization of food‐contact surfaces (Wells‐Bennik et al. 2016). Spore germination within biofilms is triggered by nutrient availability and favorable microenvironmental conditions, enabling rapid transition back to metabolically active vegetative cells (Shrestha et al. 2025). This spore–biofilm interaction creates a persistent contamination cycle that is difficult to eliminate using conventional sanitation approaches, highlighting the need for targeted control strategies.

## 3 Environmental Drivers in Meat and Poultry Processing

**FIGURE 2:**
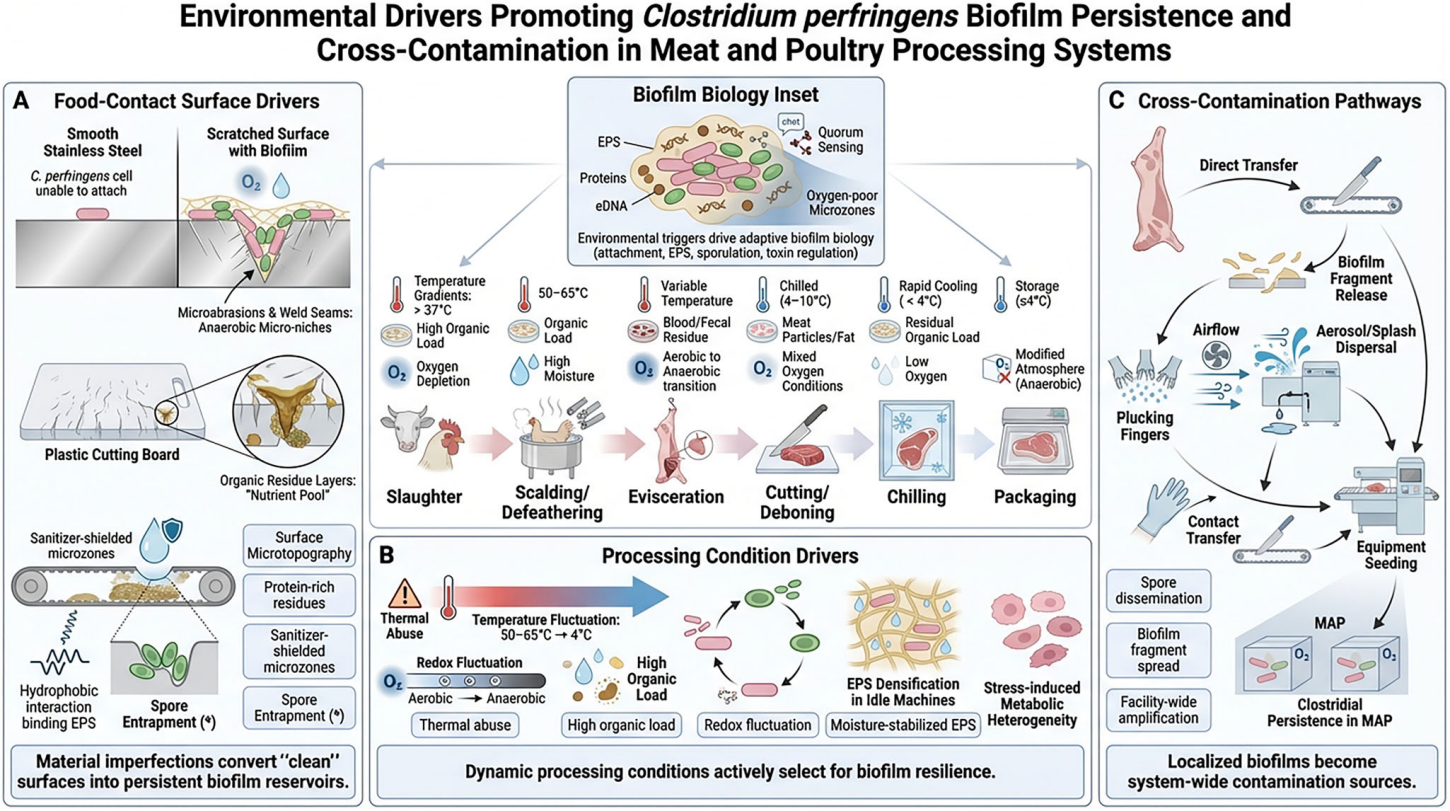
Environmental drivers promoting Clostridium perfringens biofilm persistence and cross‐contamination in meat and poultry processing systems. This schematic illustrates key environmental drivers that facilitate *C. perfringens* biofilm formation and persistence in processing environments. Surface characteristics such as roughness and the accumulation of organic residues provide protected niches for bacterial attachment and EPS production. Processing conditions including temperature fluctuations, moisture, and oxygen‐limited microenvironments further support bacterial growth and sporulation. Mechanical operations and equipment design can contribute to the dispersal of biofilm fragments and spores across processing lines, promoting cross‐contamination and recurring contamination within facilities.

Building on the biological mechanisms described above, environmental conditions in processing facilities further shape biofilm persistence and distribution. The persistence of *C. perfringens* biofilms in meat and poultry systems is fundamentally shaped by several unique environmental drivers present during slaughter, cutting, chilling and further processing (Ban‐Cucerzan et al. 2025). These drivers, including temperature abuse, nutrient availability, and fluctuating environmental conditions, create microenvironments within processing facilities where *C. perfringens* can persist and serve as a source for cross‐contamination of products. *C. perfringens* exploits the nutrient‐rich residues, anaerobic microzones, and dynamic surface product interactions that emerge throughout processing lines, enabling stable colonization and rapid regrowth even after standard sanitation cycles (Camargo et al. 2024). These conditions collectively enhance surface colonization and long‐term persistence. The interplay of these drivers is illustrated in Figure 2. Understanding these determinants is essential for designing targeted interventions.

### 3.1 Food‐Contact Surfaces

Food‐contact surfaces in meat and poultry plants serve as primary determinants of *C. perfringens* biofilm formation due to their material composition, surface microtopography, and tendency to accumulate organic residues (Alzubeidi et al. 2018). Stainless steel, while widely considered hygienic, develops microabrasions and weld seams that trap blood proteins, collagen fragments and fat droplets, substrates that not only enhance initial clostridial attachment but also support the establishment of anaerobic niches within surface imperfections (Hage et al. 2022). Plastic surfaces, such as high‐density polyethylene (HDPE) and polyurethane cutting boards, are even more vulnerable, with repeated knife scoring generating microcracks that can harbor spores and shield early biofilm microcolonies from sanitizer exposure (Bandaranayake 2024). Conveyor belts, rubber plucking fingers and scalding equipment further contribute to heterogeneous surface chemistries that enhance hydrophobic interactions and promote stable EPS anchoring (Sakhadeo and Patro 2022).

Certain surfaces act as sanitation‐resistant niches. Stainless steel weld points and surface microscratches create protected attachment sites where organic residues accumulate and oxygen diffusion is limited (Alzubeidi et al. 2018). Rubber and elastomeric components, including gaskets, seals, and plucking fingers, act as moisture‐retaining materials that facilitate spore adhesion and biofilm maturation under wet processing conditions (Ban‐Cucerzan et al. 2025). Polymeric conveyor belts further promote biofilm stability by retaining moisture, organic debris, and EPS within interlinked fibers and joints, enabling rapid recolonization following cleaning (Hu et al. 2021).

### 3.2 Processing Conditions

Processing conditions in meat and poultry plants create temporal and spatial microenvironments that strongly favor clostridial survival and biofilm maturation (Yang et al. 2024). High organic loads from blood, exudates, skin fragments, and intestinal contents provide abundant nutrients and create localized anaerobic pockets, particularly on equipment operating at warm temperatures (Grenda et al. 2023). Thermal transitions, from scalding (50–65°C) to defeathering, evisceration, cutting, and chilling, produce fluctuating redox gradients that *C. perfringens* exploits due to its ability to shift between vegetative growth, sporulation, and germination in response to minor deviations in temperature or oxygen penetration (Jia et al. 2020). Moisture‐rich areas, such as chillers, drip lines, and drainage zones, further reinforce clostridial persistence by maintaining hydration of EPS layers and facilitating rapid regrowth after sublethal sanitizer exposure (Kopper et al. 2023). Repeated contamination–cleaning cycles further reinforce microbial persistence (S. Y. Kim and Park 2023).

### 3.3 Cross‐Contamination Pathways in Slaughter and Packaging Plants

The architecture of modern meat and poultry facilities inherently promotes the spread of *C. perfringens* via multiple cross‐contamination routes, transforming localized biofilms into facility‐wide contamination hazards (Thaivalappil et al. 2025). During slaughter, carcass‐to‐equipment transfer of spores is common on dehairing rollers, plucking fingers, conveyor systems, and viscera‐handling tools, where mechanical abrasion facilitates deep microbial embedding into surface microstructures (Bottone 2010). In evisceration lines, leakage of intestinal contents introduces high loads of spores and vegetative cells that rapidly seed new biofilms, especially on damp equipment with residual organic material (Wallace et al. 2018). Real‐world surveillance and outbreak investigations in meat processing plants have reported recurrent detection of *C. perfringens* in finished poultry products, where identical strains were traced from processing equipment to retail meat samples, confirming persistent facility‐based cross‐contamination cycles (Koutsoumanis et al. 2024). During cutting, deboning, and packaging, biofilm fragments released by mechanical forces or temperature shifts can contaminate downstream products, contributing to recurrent detection of *C. perfringens* in retail meat and poultry (Priya et al. 2023). Airflow patterns, water splash zones, and worker contact further act as secondary vectors, distributing clostridial cells across distant surfaces and promoting multispecies biofilms that resist standard sanitation (Koutsoumanis et al. 2021). Among these pathways, equipment surfaces and evisceration‐related contamination represent the primary and most critical drivers of cross‐contamination, while air, water splash, and personnel act as secondary dissemination routes that amplify spread across processing lines (Reiche et al. 2025). Contaminated packaging surfaces may also act as secondary reservoirs (Mohi Alden et al. 2019).

## 4 Impact of *C. perfringens* Biofilms on Meat and Poultry Safety

The persistence and resilience of *C. perfringens* biofilms have direct implications for food safety and operational challenges in meat and poultry processing systems. Biofilm formation by *C. perfringens* in meat and poultry systems fundamentally alters the risk landscape of contamination, survival, and toxin‐mediated illness (R. Wang 2019). Unlike planktonic cells, biofilm‐embedded populations display profound shifts in physiology, stress resilience, and virulence potential, allowing them to withstand thermal treatments, evade sanitation procedures and persist throughout processing lines (Grari et al. 2025). These traits enable persistent contamination and reduced product shelf life (Basha et al. 2025). Biofilms act as reservoirs that continuously seed equipment and products. Their impacts are summarized in Table 1.

**TABLE 1:** Impact of *Clostridium perfringens* biofilms on meat and poultry safety.

Aspect	Observations	Mechanistic insights	Public health/industry implications	References
Survival under sanitation	Biofilms persist after treatment with quaternary ammonium compounds, chlorine, and heat	EPS and spore integration protect‐embedded cells	Risk of contamination in ready‐to‐eat meat	Marmion et al. (2022)
Spoilage potential	Biofilms accelerate spoilage in chilled and ambient conditions	Metabolic activity generates off‐odors, acidification	Reduced shelf life, economic loss	Mafe et al. (2024)
Toxin production	Elevated CPE and PFO in biofilm‐associated cells	Coordinated gene expression in biofilm mode	Increased foodborne illness severity	Lou et al. (2024)
Multispecies interactions	Coexistence with *Salmonella*, *Listeria*, *Campylobacter*	EPS enables spatial organization and metabolic cross‐feeding	Sanitation and pathogen control are more complex	Pant et al. (2025)
Spore‐mediated persistence	Spores embedded in EPS resist cleaning and heat	Serve as seed population for rapid recolonization	Frequent postsanitation regrowth	El‐Saadony et al. (2025)
Surface adhesion preference	Higher biofilm density on stainless steel, HDPE, rubber	Hydrophobic interactions, surface roughness	Equipment design influences contamination risk	Ciolacu et al. (2022)
Resistance to heat processing	Biofilm cells survive mild cooking conditions	Microaerophilic metabolism and EPS barrier	Potential contamination of partially cooked products	Kreling et al. (2020)
Regulatory challenges	Hard to monitor biofilm‐associated cells	Traditional CFU assays underestimate biofilm load	Underestimation may affect compliance with hygiene standards	Jo et al. (2025)

Abbreviations: CPE, *C. perfringens* enterotoxin; EPS, extracellular polymeric substances; HDPE, high‐density polyethylene; PFO, perfringolysin O.

### 4.1 Survival in Sanitation Steps and Heat Processing

*C. perfringens* biofilms can survive sanitation and heat treatments (Alzubeidi et al. 2018). EPS matrices restrict sanitizer penetration and reduce efficacy by limiting diffusion of active compounds and facilitating neutralization reactions within the biofilm matrix (Sionov and Steinberg 2022). Chemical agents such as chlorine and peracetic acid often fail to fully remove mature biofilms under high organic load or in structurally complex surfaces (A. Zore et al. 2025).

Biofilm‐associated cells and spores exhibit enhanced thermal resistance (Jaakkola et al. 2021). The EPS matrix may contribute to thermal protection by retaining moisture and creating microenvironments that reduce heat transfer; however, evidence for this mechanism in *C. perfringens* biofilms remains limited and is often inferred from studies on other biofilm‐forming bacteria (Obana et al. 2020). Heat stress may also trigger spore germination, especially during improper cooling (Cebrián et al. 2009). In meat processing systems, incomplete heating or slow cooling can therefore facilitate survival and subsequent outgrowth of spores embedded within biofilms (Alves et al. 2024). These findings indicate that conventional cooking and cooling practices may not always eliminate biofilm‐associated cells or spores, particularly under conditions of high biomass, surface protection, or inadequate process control.

### 4.2 Spoilage and Public Health Risks

Biofilms contribute to spoilage by accelerating proteolysis and producing off‐odors, particularly in low‐oxygen environments (Wagner et al. 2021). Metabolites such as fatty acids, sulfur compounds, and amines reduce product quality and shelf life (D. Wang et al. 2024). Beyond direct spoilage, biofilms play a critical role in the food industry as persistent reservoirs of contamination on food‐contact surfaces, enabling continuous release of pathogenic cells into products during processing. These surface‐associated communities are difficult to eliminate and can survive routine sanitation, thereby contributing to recurrent contamination events across production cycles (Asma et al. 2022; Sharan et al. 2022).

Biofilm cells may show increased toxin gene expression due to stress and quorum sensing (Charlebois et al. 2014). Foodborne illness associated with *C. perfringens* is commonly linked to consumption of foods containing approximately 10^5^–10^6^ CFU/g, where inadequate cooling or temperature abuse promotes rapid vegetative growth and enterotoxin accumulation (Ohnishi 2025). Contaminated products can support rapid toxin production under temperature abuse (Coppola et al. 2025). This risk is particularly significant in large‐scale foodservice operations and cooked meat products that undergo prolonged holding at suboptimal temperatures, conditions that favor both spore germination and toxin production (Maojin et al. 2025). This risk is significant in large‐scale foodservice settings. In industrial contexts, biofilms are also associated with cross‐contamination pathways, where detachment of biofilm fragments or individual cells during processing can lead to widespread distribution of pathogens across equipment, products, and processing environments (Ban‐Cucerzan et al. 2025; LaPointe et al. 2025). This phenomenon is particularly concerning for spore‐forming organisms such as *C. perfringens*, as spores embedded within biofilms can survive adverse conditions and subsequently germinate under favorable conditions, increasing the likelihood of foodborne outbreaks (Hu et al. 2021; A. Shen et al. 2019).

Furthermore, biofilm‐associated microorganisms often exhibit enhanced tolerance to environmental stresses, including disinfectants and temperature fluctuations, which complicates control strategies and increases public health risks (Omwenga and Awuor 2024). From a risk‐based perspective, the persistence of biofilm‐associated cells and spores increases the probability of recurrent contamination events, prolonged environmental survival, and consumer exposure, thereby elevating the likelihood of large‐scale outbreaks in meat and poultry processing systems (Ban‐Cucerzan et al. 2025). The persistence of such resilient microbial communities in food systems underscores their importance not only in spoilage but also in the transmission of foodborne pathogens and the occurrence of large‐scale outbreaks (Dong et al. 2025).

### 4.3 Economic and Regulatory Challenges in the Meat Industry

Biofilm formation in meat processing environments contributes substantially to increased operational costs, primarily due to the need for intensified cleaning regimes, higher sanitizer usage, and production downtime associated with sanitation procedures (Chowdhury et al. 2025; Sun, Xu, et al. 2025). Persistent biofilm‐associated contamination has been strongly linked to recurring pathogen presence, which may ultimately result in product recalls, economic losses, and disruption of supply chains (Akter et al. 2026; Sun, Xu, et al. 2025). Foodborne outbreaks and product recalls associated with contaminated meat products can generate substantial economic losses through product disposal, production shutdowns, intensified sanitation requirements, and regulatory enforcement actions, particularly in large‐scale processing facilities (Prabhukhot 2026). In addition, repeated contamination events can negatively impact brand reputation and consumer confidence, particularly in cases involving high‐profile foodborne outbreaks (Sanchez et al. 2022). Biofilms also pose significant challenges for compliance with established food safety management systems, including Hazard Analysis and Critical Control Point (HACCP) frameworks, as well as regulatory standards set by agencies such as the USDA‐FSIS and Codex Alimentarius Commission (Elafify et al. 2024). One major limitation is the inefficiency of conventional detection and monitoring approaches, as standard culture‐based sampling methods often fail to accurately capture biofilm‐associated microorganisms embedded within complex surface matrices (E. Gomes et al. 2026; Grari et al. 2025). This underestimation increases the risk of regulatory noncompliance and undetected contamination.

Furthermore, increasing regulatory emphasis on environmental monitoring programs, although often centered on pathogens such as *Listeria monocytogenes*, is also highly relevant for spore‐forming organisms such as *C. perfringens*, which can persist in processing environments and pose significant food safety risks if inadequately controlled (Bourdichon et al. 2021; Gupta and Adhikari 2022). Collectively, these challenges underscore the urgent need for improved, reliable biofilm detection methods and more effective intervention strategies tailored to food‐processing environments (X. Shen et al. 2025).

## 5 Limitations of Current Control Approaches

Conventional biofilm control strategies are insufficient for managing *C. perfringens* in meat and poultry processing environments (Sharma et al. 2025). This limitation arises because most interventions target aerobic pathogens, whereas *C. perfringens* is an anaerobic, spore‐forming organism capable of exploiting protected microniches (Ohnishi 2025). Moreover, the effectiveness of these strategies varies significantly depending on biofilm maturity, surface type and organic load, which are often not consistently addressed across studies (Rodrigues Carneiro et al. 2024). This variability has been reported across food‐processing environments, where differences in sanitation protocols and organic residues significantly influence biofilm control outcomes (add appropriate food microbiology reference). This section summarizes the major limitations of chemical, physical and biological control strategies (Renati and Madl 2025). Key limitations are presented in Table 2. Importantly, the limited success of current control approaches is largely attributed to the combined effects of EPS‐mediated protection, spore persistence, anaerobic microenvironments, and high organic loads, which collectively reduce intervention efficacy under industrial processing conditions.

**TABLE 2:** Limitations of current control approaches.

Control approach	Target	Efficacy against *Clostridium perfringens* biofilms	Reported efficacy (organism‐ and condition‐dependent)	Key limitation	Potential improvement	Evidence level (study type)	References
Chemical sanitizers	EPS and vegetative cells	Moderate to low; spore‐resistant, with reduced efficacy in mature biofilms and high organic load conditions	Reduced efficacy in mature biofilms and high organic load; limited activity against spores	Biofilm matrix shields‐embedded cells	Combine with enzymatic EPS disruptors, particularly in mature biofilms	Lab–pilot studies	Guzmán‐Soto et al. (2021), Hu et al. (2021)
Physical cleaning	Surface debris	Partial; effective on smooth surfaces but limited in microcracks and rough surfaces	Partial removal; strongly influenced by surface roughness and cleaning intensity	Mechanical removal cannot penetrate biofilm microenvironments and may cause cross‐contamination	Surface engineering, automated scrubbing to reduce contamination niches	Industrial practice	Georgakopoulos‐Soares et al. (2023), Liang et al. (2024)
Probiotics	Competing bacteria	Limited; more effective in early‐stage biofilms but restricted under anaerobic conditions	Limited efficacy; mainly effective in early‐stage biofilms under favorable conditions	EPS and redox microenvironments inhibit probiotic growth, especially in protein‐rich systems	Targeted postbiotics or bacteriocins with improved stability	Lab‐scale	Cull et al. (2022), Mazziotta et al. (2023)
Thermal treatment	Heat‐sensitive cells	Ineffective against spores; effective only against vegetative cells	Effective against vegetative cells; spores show high resistance under standard conditions	Spores survive standard cooking and may persist within biofilm structures	Higher‐temperature pulses, or combination with antimicrobials	Industrial validated	Perez‐Reyes et al. (2025)
Enzymatic treatments	EPS polysaccharides	Partial; effective against polysaccharides but protein and eDNA components remain	Variable biofilm disruption depending on matrix composition and enzyme specificity	Matrix heterogeneity and enzyme specificity reduce efficiency	Multienzyme cocktails targeting multiple EPS components	Lab–pilot studies	J. S. Kim et al. (2023), Le Sénéchal et al. (2025)
Surface coatings	Antiadhesive surfaces	Prevents initial attachment but cannot remove established biofilms	Reduces initial adhesion; minimal effect on established biofilms	Requires surface redesign and long‐term durability validation	Develop long‐lasting, food‐grade and cleaning‐resistant coatings	Pilot studies	Y. Huang et al. (2024), Uneputty et al. (2022)
Sanitizer rotation	Alternate chemical exposure	Can delay resistance development but may vary depending on rotation strategy	Variable efficacy; may delay resistance but inconsistent outcomes reported	May induce stress‐adapted phenotypes under sublethal exposure	Optimize rotation with enzymatic or physical interventions for improved efficacy	Lab–industrial reports	Arthur et al. (2024), García‐Vela et al. (2023)
Combined approaches	Multitarget (chemical + physical + biological)	More effective than single approaches, particularly when targeting EPS and spores, but still incomplete	Generally more effective than single treatments; dependent on combination strategy	Operational complexity, cost and scalability challenges	Integrate predictive hygiene and targeted multitarget interventions	Pilot–industrial	Dong et al. (2025), Qiao et al. (2025)

Abbreviations: AI, artificial intelligence; eDNA, extracellular DNA; EPS, extracellular polymeric substances.

### 5.1 Chemical Sanitizers and Their Inefficiencies

Chemical sanitizers, including quaternary ammonium compounds, chlorine, peracetic acid, and alkaline detergents, remain central to sanitation programs, yet their effectiveness against *C. perfringens* biofilms is limited (Chaves et al. 2024). Several factors contribute to this limitation. First, anaerobic microenvironments in drains, meat residues and damaged surfaces harbor metabolically inactive cells with reduced susceptibility to disinfectants (Sun, Xu, et al. 2025). Second, biofilm matrices rich in proteins and eDNA reduce sanitizer penetration and neutralize oxidizing agents (Charlebois et al. 2017). Third, repeated exposure to sublethal sanitizer levels under high organic load conditions can induce stress adaptation and increased EPS production (Kranjc et al. 2024). Importantly, studies report variable sanitizer efficacy, with oxidizing agents such as peracetic acid showing higher penetration in early‐stage biofilms, while mature biofilms under high organic load conditions exhibit significantly reduced susceptibility. This suggests that sanitizer performance is strongly context‐dependent rather than universally ineffective (Chapagain et al. 2026; Raad et al. 2026). Such variability has been reported in food‐processing systems, where organic matter and biofilm maturity significantly reduce sanitizer efficacy (Koti et al. 2026). Therefore, chemical sanitizers alone are insufficient for complete biofilm removal and should be combined with complementary strategies.

### 5.2 Physical Cleaning Limitations

Physical cleaning methods, including high‐pressure water and mechanical scrubbing, are intended to remove biomass prior to sanitation (X. Wang et al. 2020). However, *C. perfringens* biofilms frequently colonize surface irregularities such as weld joints, microcracks and conveyor interfaces, where mechanical forces are ineffective (Fernandes et al. 2024; Stine 2017). Spore formation further complicates removal, as spores can persist in crevices and germinate after cleaning. High‐pressure washing may also promote cross‐contamination by dispersing biofilm fragments across processing areas (Koutsoumanis et al. 2023). Residual moisture further facilitates rapid recolonization. While mechanical cleaning is effective for removing loosely attached biomass, its efficiency decreases substantially in mature or multispecies biofilms, particularly on damaged or porous surfaces. In some cases, aggressive cleaning may even increase contamination risk by redistributing viable cells, highlighting a trade‐off between removal efficiency and cross‐contamination potential (I. B. Gomes et al. 2021). This trade‐off has been documented in industrial hygiene studies, where improper cleaning practices contribute to pathogen spread. Importantly, repeated mechanical cleaning may progressively damage food‐contact surfaces, increasing surface roughness and creating additional attachment sites that facilitate subsequent biofilm re‐establishment. This highlights a paradox in which aggressive cleaning can simultaneously reduce surface contamination while promoting long‐term biofilm persistence (Hu et al. 2021; G.‐A. Lee et al. 2026).

### 5.3 Biological and Competitive Exclusion Barriers

Biological control strategies, including bacteriophages, competitive exclusion cultures, bacteriocins, and probiotics, are emerging alternatives (Efenberger‐Szmechtyk and Nowak 2025). However, their application to *C. perfringens* biofilms remains limited. Phages often show reduced penetration through EPS layers and decreased binding due to altered receptor expression (Waqas et al. 2023). Competitive exclusion using lactic acid bacteria is constrained by the anaerobic, protein‐rich environment that favors *C. perfringens* over LAB (Hailegebreal 2017). Bacteriocins such as nisin exhibit antimicrobial activity but are limited by matrix binding, enzymatic degradation and poor diffusion (Pan et al. 2026). Practical implementation is further constrained by industrial processing conditions (Hanson et al. 2024). Comparatively, biological approaches show greater effectiveness in early‐stage biofilms or planktonic populations, but their efficacy declines in mature biofilms due to EPS barriers and environmental incompatibility. Furthermore, inconsistencies across studies arise from differences in strain specificity, delivery methods and environmental conditions, limiting their reproducibility and large‐scale application (Al‐Madboly et al. 2024; Jones et al. 2025). In addition, much of the available evidence is derived from studies on other foodborne pathogens, and direct validation in *C. perfringens* biofilms within meat processing environments remains limited (Ban‐Cucerzan et al. 2025; Y. Kim et al. 2026). Importantly, many biological control strategies demonstrate promising efficacy under controlled laboratory conditions but show reduced and inconsistent performance in industrial environments due to complex multispecies biofilms, variable organic loads, and challenges in maintaining biological stability during processing operations (Ferré et al. 2026; X. Huang et al. 2026). Thus, although biologically derived approaches are promising, their large‐scale application remains limited. Remaining molecular and ecological knowledge gaps are summarized in Table 3.

**TABLE 3:** Future directions and research priorities.

Focus area	Current status/gap	Proposed research or innovation	Expected outcome	Evidence level	Feasibility/practical consideration	Industrial considerations	References
Standardized biofilm models	Lack of meat/poultry‐specific models	Develop industrially relevant, reproducible models	Improved reproducibility; variability reduction	Lab‐scale	Medium–high	Requires cross‐laboratory validation	Lianou et al. (2020), Sun, Yuan, et al. (2025)
Food‐grade interventions	Few validated compounds	Screen GRAS antimicrobials, enzymes, probiotics	∼2–5 log reduction	Lab–pilot	High	Approval scope and stability in food systems	Esposito and Turku (2023), Uhegwu and Anumudu (2025)
Omics‐based insights	Limited biofilm‐specific transcriptomic/proteomic data	Analyze EPS composition, stress adaptation, virulence genes	Identification of target genes	Lab‐scale	Medium	Translation to application remains limited	Dutta et al. (2024), Yuan et al. (2021)
Predictive hygiene models	Limited integration of sensor‐based monitoring and predictive models in food processing	Development of data‐driven predictive models integrating environmental monitoring data (emerging concept)	Potential reduction in contamination events	Lab–pilot	Medium	Data standardization and validation required	D. Singh (2025), Taiwo et al. (2024)
Spore‐targeted interventions	Spores are resistant to most strategies	Explore novel sporicidal enzymes or phage‐based approaches	Potential > 3 log spore reduction	Lab–pilot	Low‐medium	Regulatory approval challenges	Hwang et al. (2018), Venhorst et al. (2022)
Multispecies biofilm studies	Interactions with *Salmonella* and *Listeria* poorly understood	Coculture models to study EPS sharing and competitive dynamics	Variable outcomes; may increase resistance by 1–2 log compared to monoculture	Lab‐scale	Medium	Industrial relevance uncertain	X. Yang et al. (2023)
Economic feasibility analysis	Lack of cost‐effectiveness evaluation	Compare conventional vs. novel biofilm interventions	Cost reduction potential (∼10%–30% estimated; model‐based)	Modeling / pilot	High	Requires industry‐specific data	Cámara et al. (2022), Mathur et al. (2024)
Regulatory guidance alignment	No standard guidelines for biofilm management	Development of evidence‐based frameworks aligned with HACCP and existing regulatory systems	Improved compliance	Concept–policy level	Medium	Requires regulatory harmonization	Ban‐Cucerzan et al. (2025), Reem et al. (2025)

Abbreviations: AI, artificial intelligence; EPS, extracellular polymeric substances; GRAS, Generally Recognized As Safe; HACCP, Hazard Analysis and Critical Control Points; ML, machine learning.

## 6 Modern Strategies for Biofilm Prevention

**FIGURE 3:**
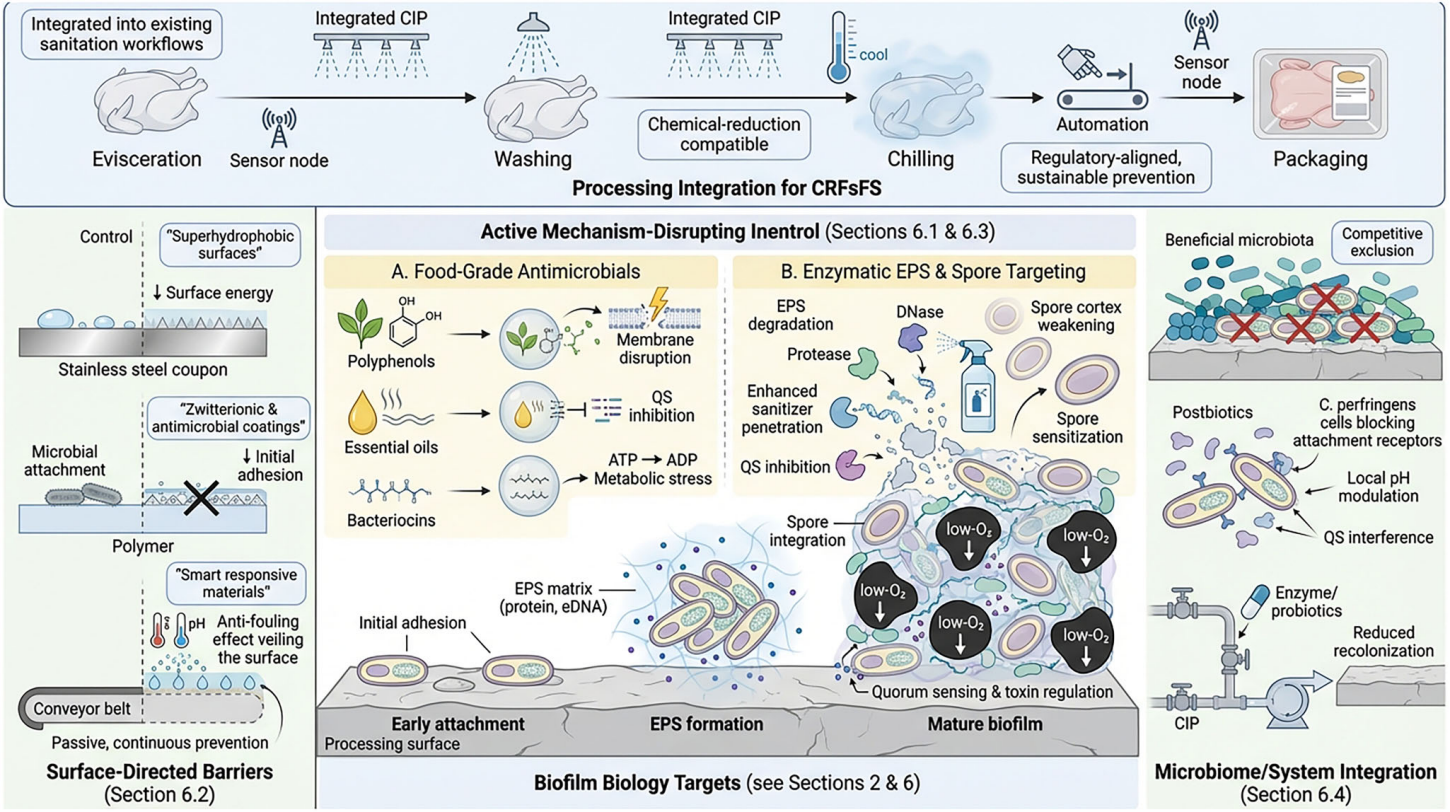
Integrated, multilayered strategies for preventing Clostridium perfringens biofilms in meat and poultry processing systems. This conceptual diagram summarizes integrated approaches for controlling *C. perfringens* biofilms in processing facilities. Surface modification strategies, including antimicrobial or antiadhesive coatings, aim to reduce initial bacterial attachment. Active interventions such as food‐grade antimicrobials and enzyme‐based treatments target EPS degradation and microbial inactivation. Biological approaches, including probiotics, postbiotics, and bacteriocins, may reduce pathogen colonization through competitive interactions. These strategies can be integrated with sanitation programs and processing‐line hygiene practices to reduce biofilm formation and minimize cross‐contamination in meat and poultry systems.

Controlling *C. perfringens* biofilms in meat and poultry systems requires approaches that move beyond conventional hygiene chemicals to mechanism‐guided, surface‐specific and ecosystem‐aware interventions (S. Singh et al. 2025). Current strategies target EPS structure, spore survival and anaerobic niche formation rather than relying solely on broad‐spectrum disinfection (Manyi‐Loh and Lues 2025). Integrated approaches combining antimicrobials, engineered surfaces, enzymatic disruption, and microbiome‐based strategies are increasingly applied (Kumar et al. 2025). An overview is presented in Figure 3. Previous reviews have largely emphasized general biofilm control strategies or focused on other major foodborne pathogens such as *Listeria monocytogenes* and *Salmonella*. In contrast, the following sections highlight intervention strategies specifically aligned with the unique physiological traits of *C. perfringens*, including its anaerobic metabolism, spore‐forming capacity, and persistence on food‐contact surfaces in meat processing environments (Al‐Madboly et al. 2024). These strategies can be broadly classified into three categories: established approaches currently used in processing environments (e.g., chemical sanitizers and CIP‐based systems), emerging strategies with demonstrated laboratory or pilot‐scale efficacy (e.g., enzymatic treatments and antimicrobial coatings) and experimental approaches that remain at early development stages (e.g., nanoencapsulation and advanced biosensing systems). Comparatively, established sanitation approaches are generally more practical and scalable for industrial implementation but often show limited effectiveness against mature biofilms and spores, whereas emerging and experimental strategies demonstrate higher target specificity and improved antibiofilm potential but remain constrained by cost, regulatory approval, stability, and limited large‐scale validation (Chowdhury et al. 2025; Sun, Xu, et al. 2025). However, the effectiveness of these strategies varies considerably depending on biofilm maturity, surface characteristics and processing conditions, and many remain at experimental or pilot scale rather than full industrial adoption.

### 6.1 Novel Antimicrobials and Food‐Grade Compounds

A growing range of food‐grade antimicrobials is being explored to control *C. perfringens* biofilms (Kao Godinez et al. 2025). Plant‐derived compounds, including polyphenols (e.g., EGCG, thymol, carvacrol), organic acids, and essential oils, exhibit antibiofilm activity by disrupting membranes, quorum sensing, and redox balance (Asghar et al. 2021). Nanoencapsulation improves antimicrobial penetration through dense EPS layers (Yan et al. 2023). Nanoliposomes, chitosan nanoemulsions, and peptide‐based nanoparticles enhance stability and controlled release under high organic load (Rousta et al. 2025). Food‐grade antimicrobials such as nisin, ε‐polylysine, and glycine esters inhibit bacterial attachment and toxin expression, supporting hurdle‐based strategies (Zeng et al. 2024). Comparatively, these compounds are more effective in early‐stage or thin biofilms, while their activity is often reduced in mature biofilms due to limited diffusion and matrix interactions. In addition, variability in reported efficacy across studies is influenced by differences in formulation, concentration and food matrix complexity (Hindieh et al. 2025). From an industrial perspective, conventional antimicrobials (e.g., organic acids, nisin) are already applied in food systems, whereas nanoencapsulation‐based delivery systems remain largely experimental due to high production cost, scale‐up challenges and regulatory approval limitations (Pateiro et al. 2021).

### 6.2 Surface Engineering and Antibiofilm Coatings

Surface engineering offers a durable strategy by targeting the interface where biofilms initiate (Zou et al. 2025). Superhydrophobic metals, plasma‐treated polymers and nanotextured surfaces reduce bacterial adhesion by modifying surface energy and roughness (Chan et al. 2021).

Antimicrobial coatings, including silver‐based films, TiO_2_ coatings and zwitterionic polymers, resist fouling and maintain activity under low‐oxygen conditions (Pereira‐Silva et al. 2025). Smart coatings capable of stimulus‐responsive antimicrobial release are emerging (Böhner et al. 2025). These systems may reduce biofilm re‐establishment after sanitation. However, their long‐term effectiveness under industrial conditions remains uncertain, as coating durability, repeated cleaning cycles and potential regulatory limitations may affect performance. Compared to chemical sanitizers, these approaches offer preventive rather than immediate removal effects, and their cost‐effectiveness at scale is still under evaluation (Abi and Banat 2026; Elbehiry and Alajaji 2026). In practice, most surface engineering approaches are currently at pilot or experimental stages, with limited large‐scale implementation due to equipment modification requirements and regulatory approval barriers (Nanthini et al. 2026). In addition, the high cost of material fabrication, maintenance, and retrofitting of existing processing equipment may limit industrial adoption, particularly for small‐ and medium‐scale processors (Qureshi et al. 2026). Regulatory approval and safety assessment of nanomaterials used in food‐contact surfaces also remain significant challenges, as concerns related to nanoparticle migration, long‐term stability, and potential toxicity require further validation before widespread commercial application (Schoonjans et al. 2023).

### 6.3 Enzymatic Disruptors Targeting EPS or Spores

Enzymatic approaches disrupt biofilms by degrading matrix components of *C. perfringens* (J. S. Kim et al. 2023). Proteases, DNases and amylases break down structural macromolecules, improving sanitizer penetration. Mechanistically, proteases hydrolyze structural proteins involved in cell adhesion and matrix stability, DNases degrade eDNA that contributes to biofilm cohesion and structural integrity, while amylases hydrolyze polysaccharide components that form the EPS scaffold (Scalia and Najmi 2025). Together, these enzymatic actions weaken biofilm architecture, enhance matrix disruption, and increase exposure of embedded cells and spores to sanitizers (Al‐Madboly et al. 2024). Certain formulations retain activity under high organic load conditions (Subirade et al. 1998). Spore‐targeting enzymes, including cortex‐lytic enzymes, further enhance control (Zhang, Gong, et al. 2025). These enzymes act by degrading the protective cortex layer surrounding spores, thereby promoting spore germination or increasing susceptibility to heat and chemical disinfectants (Koopman et al. 2022). These systems can be integrated into CIP processes or applied directly to surfaces. In comparison to conventional sanitizers, enzymatic treatments are more effective as complementary strategies rather than standalone solutions, as they primarily weaken biofilm structure without fully eliminating viable cells. Their performance is also influenced by enzyme stability, temperature and pH conditions, leading to variability across studies (Costa et al. 2026). Industrially, enzymatic approaches are considered promising but remain in early adoption stages, with challenges related to cost, stability during repeated CIP cycles and compatibility with existing sanitation protocols (Nagay et al. 2025).

### 6.4 Probiotics, Postbiotics, and Bacteriocins

Microbiome‐based approaches offer biological control of *C. perfringens* biofilms. Probiotic strains (e.g., *Lactobacillus plantarum*, *Lb. reuteri*, *Bacillus* spp.) inhibit attachment through acidification, biosurfactant production, and competitive exclusion (Aziz et al. 2024). However, most of these mechanisms have been demonstrated in other foodborne pathogens, and direct evidence for their effectiveness against *C. perfringens* biofilms in meat processing environments remains limited (Oliulla, Mizan, Kang, et al. 2024). Postbiotics provide stable alternatives by delivering bioactive metabolites without live cells (Rafique et al. 2023). While postbiotic activity has shown promising antibiofilm effects in general biofilm systems, their specific impact on *C. perfringens* biofilms has not been extensively validated and is often inferred from studies on other Gram‐positive bacteria (Bhattrai et al. 2026). Bacteriocins such as nisin and pediocin exhibit strong activity against vegetative cells and inhibit early biofilm formation (Darbandi et al. 2021). Among these, nisin has demonstrated activity against *C. perfringens* vegetative cells; however, its efficacy against established biofilms is variable and influenced by matrix composition and environmental conditions. Encapsulation improves stability under processing conditions (Xu et al. 2024). These strategies serve as complementary tools to conventional sanitation. Nevertheless, their effectiveness is highly context‐dependent, with reduced activity observed in protein‐rich, anaerobic environments typical of meat processing systems. In contrast to laboratory findings, competitive exclusion is often inconsistent under industrial conditions due to environmental variability and strain‐specific interactions (Karbowiak et al. 2023). In real meat processing systems, additional limitations include reduced survival and metabolic activity of probiotic strains during cold storage, interference from indigenous microbiota, instability of bioactive compounds under processing stresses, and challenges in maintaining consistent efficacy across different product matrices and sanitation conditions (Nascimento and Barros 2025). Overall, while microbiome‐based strategies show promise, their application to *C. perfringens* biofilms in real processing environments remains insufficiently characterized, and further organism‐specific validation is required. Currently, bacteriocins such as nisin are commercially used, whereas probiotic and postbiotic applications in meat processing environments remain largely experimental due to regulatory and stability concerns (Wu et al. 2023).

### 6.5 Emerging Monitoring Technologies

Emerging technologies enable early detection of biofilms in processing environments. Fluorescence imaging, hyperspectral analysis, and biosensors allow rapid, nondestructive detection of contamination on food‐contact surfaces (A. Lee et al. 2021; Sun, Yuan, et al. 2025). These tools help identify contamination hotspots before mature biofilms develop (Akdemir Evrendilek 2026).

Molecular methods, including PCR‐based assays and sensor platforms, provide rapid detection of *C. perfringens* without culture‐based delay (Anil et al. 2026; J.‐H. Kim and Oh 2021). These approaches support the transition toward proactive monitoring systems. Comparatively, fluorescence imaging and hyperspectral systems are advantageous for rapid surface screening and real‐time visualization but may show lower organism specificity, whereas PCR‐based and biosensor platforms offer higher sensitivity and specificity for pathogen detection but often require greater technical expertise, sample preparation and higher operational costs (Oliveira et al. 2025). However, while these technologies improve detection sensitivity, their implementation in industrial settings is often limited by cost, requirement for technical expertise and integration with existing hygiene management systems. Furthermore, detection does not equate to control, and their effectiveness depends on how rapidly corrective actions are implemented (Akinbamini et al. 2025). Most advanced sensing and artificial intelligence (AI)‐integrated monitoring systems remain at experimental or pilot scale, with limited adoption in routine meat processing due to cost and infrastructure requirements.

## 7 **Future Perspectives for Controlling** *Clostridium perfringens* **Biofilms in Meat Processing**

Future research should prioritize practical strategies to prevent and monitor *C. perfringens* biofilms in meat and poultry processing environments. Although substantial progress has been made in understanding biofilm biology, several operational challenges remain that require investigation under realistic industrial conditions (Shokeen et al. 2020). First, the development of biofilm‐resistant food‐contact surfaces represents an important preventive strategy for the meat industry. Novel surface coatings, antiadhesive materials and modified stainless‐steel finishes have shown promise in reducing bacterial attachment and delaying biofilm maturation on processing equipment (Jindal et al. 2016). Future studies should evaluate these materials under commercial processing conditions, including repeated cleaning cycles, mechanical wear, and exposure to organic matter, to determine their durability and feasibility for large‐scale industrial adoption.

Second, rapid detection and monitoring technologies are needed to identify early biofilm formation in processing facilities. Emerging biosensors, molecular detection platforms and real‐time environmental monitoring systems may enable processors to detect contamination hotspots before mature biofilms develop (Ban‐Cucerzan et al. 2025). Integration of such tools into routine sanitation verification programs could improve early intervention and support risk‐based monitoring in meat processing plants. In addition, future systems integrating AI, machine learning, and smart sensor networks may enable predictive monitoring of biofilm development, allowing processors to identify high‐risk contamination zones before persistent biofilms become established.

Third, sporulation dynamics within biofilms require greater attention. Because *C. perfringens* spores exhibit high resistance to environmental stresses and sanitation treatments, understanding how spores interact with the biofilm matrix may help explain the persistence of contamination in processing environments (Shahid et al. 2025). Future research should focus on identifying sanitation strategies that effectively target both vegetative cells and spores embedded within biofilm structures. Advanced molecular approaches, including transcriptomics, proteomics and metabolomics, may further improve understanding of the regulatory pathways linking sporulation, EPS production and stress adaptation within biofilms.

Finally, future control strategies should emphasize multihurdle sanitation approaches that integrate physical cleaning, optimized chemical disinfectants and biological antimicrobials. Combining complementary interventions may enhance biofilm removal efficiency while reducing the reliance on harsh chemical treatments and supporting more sustainable sanitation practices in meat processing facilities. Emerging technologies such as nanoencapsulated antimicrobials, enzyme‐functionalized coatings, and stimulus‐responsive antimicrobial surfaces may provide next‐generation approaches for targeted biofilm disruption under industrial conditions.

From an industrial perspective, implementing these strategies will require collaboration between researchers, equipment manufacturers, and food processors. Advances in equipment design, hygienic surface engineering, real‐time contamination monitoring, and scalable sanitation technologies will be critical for translating laboratory findings into practical solutions that improve food safety and operational efficiency in the meat and poultry industry. Importantly, future research should increasingly prioritize pilot‐scale and commercial validation studies to bridge the gap between laboratory efficacy and industrial applicability of antibiofilm interventions.

## 8 Literature Search Strategy and Selection Criteria

This review was developed using a structured literature search approach to ensure comprehensive and balanced coverage of current knowledge on *C. perfringens* biofilm formation, persistence, and control in meat and poultry processing environments. Relevant studies were identified through searches of major scientific databases, including Web of Science, Scopus, PubMed, and ScienceDirect. The search focused on publications from 2015 to 2025, with particular emphasis on recent studies to capture advances in biofilm research and control strategies. Search terms included combinations of the following keywords: “*C. perfringens*,” “biofilm,” “meat processing,” “poultry processing,” “food‐contact surfaces,” “spores,” “antimicrobial resistance,” and “biofilm control.” Studies were selected based on their relevance to food systems, especially those addressing biofilm formation, persistence, and control in processing environments. Additional consideration was given to studies providing mechanistic insights or industrial applicability. Non–peer‐reviewed sources, duplicate records, and studies not directly related to food‐processing contexts were excluded. The selected literature was critically evaluated and synthesized to identify key trends, limitations, and knowledge gaps, with emphasis on organism‐specific evidence and practical relevance to meat and poultry processing systems.

## 9 Conclusion

*C. perfringens* biofilms represent an important but often under‐recognized challenge in meat and poultry processing environments. Their ability to persist through spore formation and biofilm development enables survival under sanitation stress and facilitates repeated contamination of food‐contact surfaces. These characteristics complicate conventional control strategies and increase the risk of product contamination and foodborne illness. Current control approaches, including chemical disinfectants, physical treatments, and biological interventions, can reduce biofilm formation but may be limited by the structural complexity and resilience of biofilm communities. Improving sanitation effectiveness therefore requires a better understanding of the ecological and physiological factors that support biofilm persistence in meat processing environments. Future efforts should focus on integrating improved surface sanitation, targeted antimicrobial strategies and early monitoring systems within existing food safety management programs. Strengthening these approaches will be critical for reducing *C. perfringens* contamination risks and improving the microbiological safety of meat and poultry products. Importantly, effective long‐term control of *C. perfringens* biofilms will require a shift from conventional reactive sanitation toward integrated, mechanism‐based and risk‐oriented prevention strategies that specifically target sporulation, EPS‐mediated protection and environmental persistence within processing systems. Furthermore, translating laboratory‐scale findings into commercially applicable interventions remains essential for improving industrial sanitation efficiency, reducing food safety risks and supporting sustainable meat production practices.

## Author Contributions

**Md Anamul Hasan Chowdhury**: software, data curation, formal analysis, writing – review and editing, writing – original draft, investigation, conceptualization. **Chowdhury Sanat Anjum Reem**: writing – original draft, methodology, software, data curation. **Md. Ashrafudoulla**: investigation, validation, data curation, formal analysis. **Hae Jung Yoon**: investigation, supervision. **Sang‐Do Ha**: investigation, conceptualization, funding acquisition, project administration, supervision.

## Funding

This work was supported by the Chung‐Ang University Young Scientist Scholarship (CAYSS 2024); and the Chung‐Ang University research grant in 2025.

## Conflicts of Interest

The authors declare no conflicts of interest.
